# Stereological analysis of cholinergic neurons within bilateral pedunculopontine nuclei in health and when affected by Parkinson's disease

**DOI:** 10.1111/bpa.70011

**Published:** 2025-04-15

**Authors:** Puneet Kumar Sharma, Steve Gentleman, David Trevor Dexter, Ilse Sanet Pienaar

**Affiliations:** ^1^ Department of Brain Sciences Imperial College London London UK; ^2^ Department of Neurosurgery Imperial College Healthcare Trust London UK; ^3^ Parkinson's UK London UK; ^4^ School of Biomedical Sciences College of Medicine and Health, University of Birmingham Birmingham UK; ^5^ Centre for Human Metabolomics North‐West University Potchefstroom South Africa

**Keywords:** 3‐D reconstruction, cholinergic neurons, cytoarchitecture, deep brain stimulation, Parkinson's disease, pedunculopontine nucleus, rostro‐caudal axis

## Abstract

During Parkinson's disease (PD), loss of brainstem‐based pedunculopontine nucleus' (PPN) cholinergic neurons induces progressive postural‐gait disability (PGD). PPN‐deep brain stimulation inconsistently alleviates PGD, due to stereotactic targeting inaccuracies resulting from insufficiently detailed human PPN anatomical descriptions. Relatedly, rodent studies show rostro‐caudal clustering of PPN‐cholinergic neurons, reflecting functional sub‐territories. We applied unbiased cerebro‐bilateral 3‐dimensional (3‐D) stereology to post‐mortem PPNs from PD versus neurological‐control cases, to estimate total numbers of cholinergic neurons and describe their rostro‐caudal distribution. Given ambiguous descriptions of the PPN's confines, we utilized two complimentary definitions of the PPN's anatomical boundaries. The first was based on the structure's gross anatomy, by considering the nucleus as a recognizable “channel” enclosed by distinct white matter fiber tracts (WMFT) encompassing the medial lemniscus, central tegmental tract and decussation of the superior cerebellar peduncle. Second, the PPN was recognized by its histological architecture, as a dense collection of cholinergic neurons (the “Ch5” group) that were immunoreactive for choline acetyltransferase (ChAT), the enzyme responsible for biosynthesis of the neurotransmitter acetylcholine. Many such ChAT‐immunoreactive neurons were dispersed within the traversing tracks and hence the PPN's Ch5‐based outlining method permitted their stereological capture while also allowing distinction between the PPN's two subnuclei, namely the pars compacta (PPNc) and pars dissipata (PPNd), based on subnuclei‐specific cholinergic cytoarchitectural organization. We further reconstructed template data as 3‐D renders, revealing gross morphological differences between control and PD‐affected PPNs. PD brains revealed significant PPN cholinergic neuronal loss, particularly affecting the PPNd. Control cases showed bimodal clustering of cholinergic neurons, prominently affecting left‐sided PPNs. Most PD cases revealed more severe cholinergic neuronal loss in right‐sided PPNs, potentially driving symptom lateralization. Our study provides a comprehensive cholinergic cytoarchitectural atlas of the human PPN in health versus during PD.

## Graphical abstract

(A) PPN‐cholinergic neurons degenerate during PD. We stereologically counted their rostro‐caudal distribution in post‐mortem PPNs in health versus PD. (Bi) Serial PPN‐containing samples were cholinergic antibody‐DAB stained. (Bii) Only DAB‐stained neurons on green lines and inside the box were counted. (Ci) PD versus control neuronal counts when outlining the PPN via its cholinergic neurons or (Cii) WMFTs. (Ciii) PPNc versus PPNd neuronal counts in control versus PD‐affected brains.

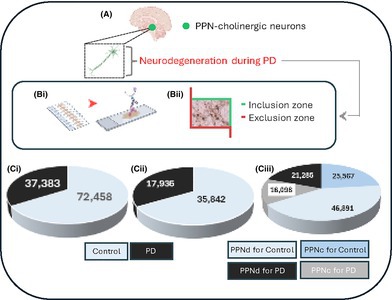

## 1 INTRODUCTION

Attempts have been made to improve stereotaxic accuracy of a neurochemically and functionally heterogeneous structure termed the pedunculopontine nucleus (PPN), which is based at the junction between the midbrain and pons, to form an elongated neuronal collection along the rostro‐caudal axis that is anatomically subdivided into a rostral and a caudal portion [1]. Although consensus is lacking as to the PPN's exact anatomical localization, there is general acceptance that the PPN locates to within the caudal pontomesencephalic tegmentum in the upper brainstem and lies caudal‐inferior to the red nucleus (RN), dorsal to the substantia nigra (SN), lateral to the medial lemniscus (ML) and medial to fibers of the decussation of the superior cerebellar peduncle (SCP) [2]. The PPN principally contains cholinergic, glutamatergic and γ‐aminobutyric acid (GABA)ergic neurons [3, 4, 5], with several post‐mortem studies which demonstrated that the PPN undergoes substantial neurodegeneration affecting the cholinergic neurons during Parkinson's disease (PD) [4, 6, 7, 8, 9, 10, 11, 12, 13, 14, 15], summarized in Table S1. Studies that applied stereology‐based principles quantified ~38% cholinergic neuronal loss in PD‐affected post‐mortem brains [4, 11, 13]; this cellular loss is believed to underlie posture and gait disorder (PGD) that frequently results in falls in patients and animal models of Parkinsonism [16, 17, 18, 19, 20]. Moreover, these symptoms remain unresponsive to conventional dopamine replacement therapy provided as Levodopa medication, highlighting the fact that disruption in other neurotransmitter systems may contribute to PD‐related extrapyramidal symptoms [21].

The PPN's importance in PD pathogenesis was further emphasized in studies that highlighted the potential benefits of targeting the PPN via deep brain stimulation (DBS). PPN‐DBS showed effective amelioration of PD‐related PGD; however, clinical trials demonstrated mixed results [22, 23, 24, 25, 26, 27, 28, 29, 30]. Important contributing factors that may explain such variable results include patient selection criteria, subjective differences in self‐report measures for evaluating the symptom‐relieving effects of PPN‐DBS, small case numbers, stimulation protocol variance across neurosurgical centers, and co‐existing patient morbidity. Technical difficulties for precisely targeting the PPN likely also play a role in the significant variance relating to the reported clinical benefits following PD patients receiving PPN‐DBS [31]. In this regard, brainstem anatomy is renowned for showing significant inter‐subject variation and not lending itself well to the application of standard reference points such as the anterior commissure–posterior commissure line [2, 14, 32].

Attempts have been made to improve stereotaxic accuracy for intra‐PPN‐DBS electrode implants by fusing magnetic resonance imaging (MRI) data with positional anatomical information from a brain atlas [33]. However, such efforts are limited by the accuracy of the original atlas descriptions, none of which were designed for targeting the PPN specifically. In fact, only a single human brain atlas, compiled by Paxinos and Huang in 1995 [34], labels the PPN on consecutive brain sections. However, this atlas derives from the anatomical brain dissection and subsequent histological staining of brain tissue sections taken from a single 59‐year‐old neurologically healthy male, raising questions about the manual's translational value relative to an older PD‐affected clinical population. Another human brain atlas was compiled by Schaltenbrand and Wahren [19] but labels the PPN on only a single brain section. As such, neurosurgeons targeting the PPN via stereotaxic means are guided by very limited information which may not generalize well across different patients. Moreover, Agostinelli and others [35] utilized automated segmentation to demonstrate the distribution of cholinergic, serotonergic and catecholaminergic brainstem neurotypes in spatial relation both to each other and to an important spatial reference, the cerebellum. This histological axial‐level data was paired with 7‐Tesla MRI imaging performed on a cadaveric male human brainstem and cerebellum, allowing for correlation between the chemoarchitecture and corresponding MRI. The study demonstrated the feasibility by which PPN neuronal groups can be identified from MRI scans, opening the possibility for aiding more precise DBS electrode placement to optimize clinical benefit from PPN‐DBS.

The need for stricter precision in neurosurgical targeting of the PPN was highlighted by Zitella and colleagues [31], who reported that millimeter‐scale differences in target acquisition significantly alter neural target stimulation, with profound implications for DBS‐mediated management of PD's clinical symptoms. This raises the possibility that existing human brain atlases, being fundamental reference tools for targeting deep‐lying brain structures such as the PPN, may be unsuitable for determining the PPN's location in PD patients. That is, such reference tools do not account for factors inherent to the PPN's cellular organization that could influence effective delivery of PPN‐DBS. For instance, although several studies performed on rodent brains demonstrated anatomical clustering of neurotypes within the PPN, possibly underpinning region‐specific functional specialization [34, 36, 37, 38], no study thus far has reported on the presence of similar neuronal density clusters in the human PPN, let alone on the effects of neurodegenerative disease on such cellular organization patterns. The latter aspect holds consequences for patients' functional recovery prognosis following PPN‐DBS, where placing a DBS electrode's tip within a target area of intact neuroarchitecture (such precise targeting being aided by guides reporting on the extent and spatial distribution of PPN neuronal loss) as opposed to a microlesion location where neuronal somas have degenerated greatly improves expected long‐term clinical outcomes. Sparse data also exist on the symmetry of the PPN across the midline, and the question of whether it is preserved or altered by neurodegenerative diseases such as PD.

Taken together, this highlights a need for developing optimized approaches to define the PPN's boundaries to accurately target the PPN when affected by conditions such as PD that impact on its structural integrity. Here, using combined immunohistochemistry (IHC) and stereology, we provide an accurate account of the number and distribution of the PPN's cholinergic neurons present in post‐mortem brains of PD patients (≥Braak stage III), compared to neurological‐control PPNs. Quantifications were made across the midline to ascertain whether there were significant laterality differences that may correspond to phenotypic variables such as handedness or asymmetric symptom progression, which could provide a simple, yet essential refinement for PPN‐DBS targeting. Our findings demonstrate the presence of rostro‐caudal clustering of cholinergic neurons, which may outline functional sub‐territories of the PPN. We also explore the extent to which this neuronal subtype interdigitates with traversing white matter fiber tracts (WMFT), conventionally used to define the PPN's boundaries. This has important consequences for the neural population count attributable to the PPN, and for targeting the nucleus using existing stereotaxic atlases, as this risks missing the section of the nucleus that lies outside these WMFT boundaries. Finally, we demonstrate that the PPN's position can reliably be ascertained from the position of the inferior colliculus (IC), the trochlear nucleus and the tegmental dimensions, anatomical structures that are visible in standard MRI conditions and in histologically stained brain tissue sections.

## 2 MATERIALS AND METHODS

### 2.1 Subjects, brain tissue acquisition and brain block sectioning

PPN‐containing human brain tissue blocks from four neurological‐control subjects and four PD patients were prospectively acquired from the Parkinson's UK Brain Bank at Imperial College London. Control cases were free from significant neuropathology; PD‐confirmed cases displayed the following neuropathological features upon autopsy: Severe atrophy of the darkly pigmented areas seen in hematoxylin and eosin (H&E)‐stained coronal sections taken at the level of the substantia nigra pars compacta (SNpc) and locus coeruleus (LC), correlating with the death of the “A9” dopaminergic and noradrenergic neurons in the SNpc and LC, respectively [39], and presence of cytoplasmic deposits within neuronal cell bodies (“Lewy” bodies) and dystrophic neurites (“Lewy” neurites) that were immunoreactive for the protein α‐synuclein [40]. Table 1 provides a summary of the demographic and clinical characteristics of the control and PD subjects included in this study.

**TABLE 1:** Clinico‐demographic characteristics summary of neurological controls and PD cases from which post‐mortem tissue was derived.

Case identifier	Cerebral hemisphere	Sex	Age at death (in years)	Cause of death
C1	R & L	M	76	CD
C2	R	F	97	LRTI
C3	R & L	F	83	CD
C4	R & L	F	95	CD

		1:3 (M:F)	88 ± 5	
PD1	R & L	F	69	CD
PD2	L	F	92	CD
PD3	R & L	M	87	CD
PD4	R & L	M	80	CD
		2:2 (M:F)	82 ± 5	

*Note*: Where applicable, summary results are shown as mean ± SD.

Abbreviations: CD, cardiovascular disease; F, female; L, left; LRTI, lower respiratory tract infection; M, male; R, right.

PPN‐containing left–right hemispheric‐blocks were available as case pairs from three neurological control and three PD patients; for one control case, only the right‐sided PPN‐containing brain tissue block was available, while for one PD case, only the left‐sided PPN was available. Brainstem blocks containing the entire PPN were dissected as single pieces at the level of the caudal IC through to the rostral opening of the 4th ventricle, as described [9, 15, 41]. Dissected autopsied tissue was fixed in 10% formalin solution for at least 2 months and then embedded in paraffin. The fixed brain tissue blocks were serially sectioned at 20 μm thickness using a microtome (Leica, Germany), floated on water in a bath set at 40°C, then mounted onto Superfrost‐Plus slides (Menzel‐Gläser, USA). Slides were dried completely before storing at room temperature (RT) until histological processing. Every section was retained, from the beginning to the end of a sampled tissue block.

### 2.2 Immunohistochemical staining of the sections and anatomical identification of the PPN

**FIGURE 1:**
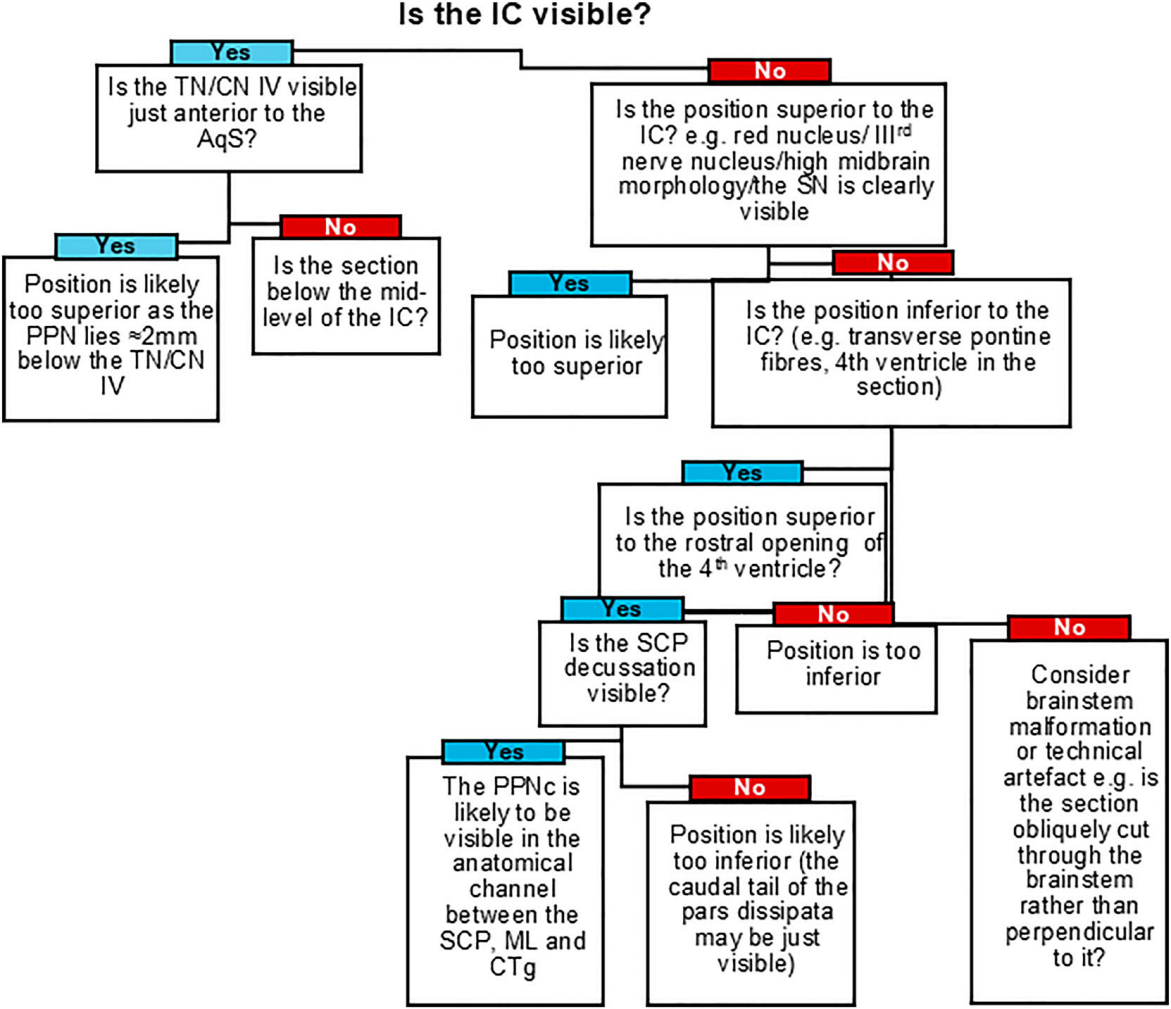
A depiction of the workflow for confirming the presence and delineating the PPN for subsequent cell type‐specific stereological quantifications. The schema allowed for predicting the presence of the PPN's Ch5 cholinergic neurons within the rostral brainstem, through stepwise identification of anatomical landmarks that were visible when viewing unstained brain sections under a low‐powered (5× air‐immersion) microscope objective lens. CN, cranial nerve; M‐L, mediolateral; ML, medial lemniscus; PPNc, pedunculopontine nucleus pars compacta; SCP, superior cerebellar peduncle decussation; SN, substantia nigra; TN, trochlear nucleus.

We applied a two‐fold approach to identify the PPN and determine its outer boundary. The human PPN is often defined based on its distinct surrounding WMFTs, that is, decussation of the SCP that forms its superior‐medial margin, the central tegmental tract (CTg) that forms its inferior‐medial limit, and the ML that forms the PPN's lateral boundary [33, 42, 43]. This anatomical description describes the PPN as lying within an anatomical “channel” located between these identifiable WMTFs. For this method, we defined the PPN by means of its surrounding WMFTs by using templates drawn from tissue sections stained with Luxol fast blue (LFB) to differentiate white‐ from grey matter, and counterstaining with cresyl fast violet (CFV) to identify neuronal groups (nuclei). A predictive key (Figure 1) was developed to reliably predict the presence of PPN‐cholinergic neurons in sectioned tissue containing the rostral pontine region, based on the visibility of key anatomical landmarks under low‐powered (5× air‐immersion objective lens) microscopy of such LFB‐CFV stained tissue sections.

**FIGURE 2:**
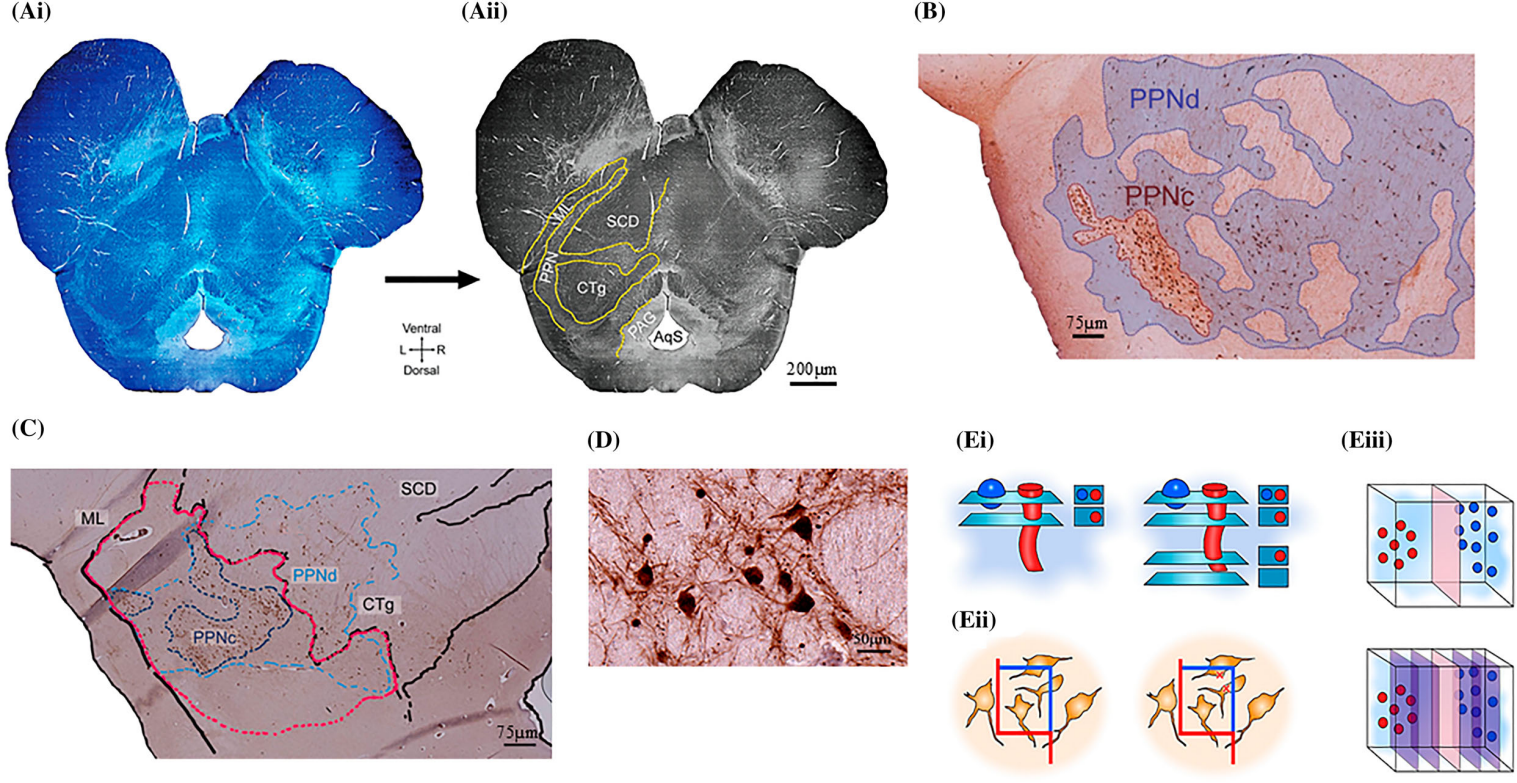
Workflow depicting dual boundary definitions applied to the PPN. The PPN shows poorly defined neuronal clustering, rendering setting reproducible anatomical boundaries to facilitate cross‐sample comparisons challenging. (Ai, ii) Juxtaposed grey matter‐white matter tracts identified via LFB staining provided anatomical boundaries; CFV counterstaining facilitated interpretation of gross morphology. (Ai) Each LFB‐stained tissue section was tile‐scanned, then digitally assembled (“stitched”) into a larger mosaic image. (Aii) The subsequent image was digitally converted to a grey‐scale image and contrast enhanced using Photoshop software. Grey‐white matter boundaries were manually traced (yellow lines) with a digital tablet and pen. The AqS, CTg, ML, PAG and SCP comprised the PPN's white matter boundaries. The template layer was digitally superimposed on mosaic images of tissue sections IHC‐stained for the PPN neuronal types. (B) Another method delineated the PPN based on its IHC‐stained “Ch5” cholinergic population, allowing for a PPNc‐PPNd distinction. In this enlarged image of the PPN region, the PPNd (transparent blue overlay) is portrayed as an irregular region containing neurons interdigitating with traversing WMFTs and showing no clear boundary. (C) The digital template was superimposed onto corresponding ChAT IHC‐stained sections, enabling consistent PPN boundary delineations to facilitate control versus PD cross‐sample comparisons for cholinergic neuronal stereological counts. Dotted lines delineate white matter tract boundaries. Atlas data [19, 34] portrays the PPN as a rostral brainstem region lying between an anatomical channel (C, red outline), shown in this ChaT‐stained brain tissue section from a control case; the CTg, SCP and ML aids orientation. The PPNc (dark blue outline) is discerned from the PPNd (light blue outline) based on cholinergic neuronal density and non‐transgression of WMFT boundaries. The PPNd contains ChAT‐ir neurons that widely distribute within the surrounding white matter. (D) A representative section for ChAT‐ir neurons of the PPN in a neurological‐control case scanned at high‐powered (using a 20× objective lens) resolution. (E) Illustrates the main issues in sectional counts that are overcome by applying stereological principles. (Ei) A count made over two sections would produce the false result of three objects, as the red object is counted twice. By introducing a separate reference frame, stereology correctly quantifies the total number of objects as two, by discounting any object seen in both the sampling and reference frames. (Eii) Illustrates the practical use of counting frames; only cells within the counting frame, or crossing the blue but not the red, are marked and counted. This prevents counting cells twice which lie across sampling frames in a single section. (Eiii) Illustrating the concept of systematic sampling, whereby clustering of neurons might result in falsely high or low estimates of the total population, depending on how the sample is divided. Systematic sampling across the entire ROI mitigates against the effect of clustering by sampling more consistently throughout.

In addition, we used an alternative PPN delineation method, comprising of plotting the PPN by means of the distribution of the “Ch5” group of cholinergic neurons [33], used frequently for outlining the rat PPTg [3, 34, 36, 37, 44]. The IHC‐staining using a cholinergic antibody marker revealed cholinergic neurons containing large somas that were distinct from another brainstem cholinergic nucleus, namely the latero‐dorsal tegmental nucleus (LDT) (cholinergic cell group “Ch6”) [1]. Although the use of the grey‐white matter‐based anatomical definition of the PPN is highly reproducible and excludes reliance on cellular distribution patterns, it inherently fails to include neurons that lie within WMFTs traversing the PPN. Hence, the second anatomical definition that we applied to the PPN aimed to mitigate against the risk of inadvertently excluding these PPN‐resident neurons from the neuronal count by providing a broader template for the PPN region, as a guide for including neurons that interdigitate the WMFTs. Moreover, the “Ch5”‐based method allowed us to draw anatomical distinction between the PPN's two main sub‐regions, namely the pars compacta (PPNc) and pars dissipata (PPNd), the PPNc being notable for its dense clustering of the “Ch5” cholinergic cell group that sends ascending projections to the thalamus [1], while the PPNd's cholinergic neuronal population is significantly sparser and hence somas are more dispersed. Figure 2 depicts the workflow for the dual boundary definitions as was applied here to the PPN.

The brain tissue sections, collected serially at evenly spaced intervals (120 μm), allowed adequate spacing to avoid duplicate counting of the same cholinergic neurons stained in the preceding section per series, that is, in every group of six contiguous sections, were stained for LFB‐CFV using the first‐in‐series section, with the second‐in‐series section that was chromogen stained for a cholinergic marker. Specifically, the complete, sequentially arranged series of sections was divided into groups of six, spanning the rostro‐caudal extent of the tissue block. Every 6th section was treated with the same IHC stain. Thus, sections 1, 7, 13, etc. were treated with LFB‐CFV, while sections 2, 8, 14, etc. were stained with the cholinergic cellular marker for mapping the cholinergic neuronal distribution to the WMFT anatomical structural information provided by the LFB stain. The remaining sections were used for optimizing staining protocols.

The sectioned tissue's wax was softened by placing it in a 60°C oven for 1 h, then deparaffinized by immersing it in xylene (MD Biosciences, Switzerland) for 3 × 5 min, cleared in 100% methylated spirits (Sigma‐Aldrich, USA) for 3 × 5 min, before hydrating through a descending order of absolute ethanol (EtOH, 2× 100% > 90% > 70% > 50%) for 5 min each, followed by immersion in distilled water for 5 min. For the LFB‐CFV stain, we followed our published protocol [45]. The cholinergic stain consisted of peroxide quenching by incubating the sections in 0.3% hydrogen peroxide (H_2_O_2_, Sigma‐Aldrich, UK), diluted in phosphate buffered saline (PBS) for 20 min at RT. Antigen retrieval was performed by immersing the sections in 10 mM sodium citrate buffer (pH 6) and heating them in a steam cooker for 20 min, after which the sections were washed and cooled to RT under running tap water. The sections were then incubated overnight at 4°C with the goat polyclonal primary antibody (1:300, #AB144P, Millipore) for labelling cholinergic neurons by recognizing choline acetyltransferase (ChAT), the enzyme that catalyzes the resynthesis of acetylcholine (ACh). After overnight incubation, the samples were washed well with PBS before applying biotinylated horse (anti‐goat) IgG secondary antibody (1:200, #BA‐9500, Vector Laboratories) for 2 h at RT, and then washing with PBS. The samples were incubated in avidin‐biotin complex solution (Vector Laboratories, UK) for 30 min at RT, washed with PBS and then applying DAB (3,3′‐diaminobenzidine; Vector Laboratories) chromogen for ~10 min to visualize the immunoreaction. After immersing the slides in distilled running water for several minutes, the sections were dehydrated in an ascending series of EtOH (70%; 90%; 2× 100%), cleared in xylene, before mounting under coverslips with dibutylphthalate xylene (DPX, Sigma‐Aldrich) and cured for 24 h before inspection.

LFB‐CFV stained sections were digitally scanned as tiled mosaics using a 10x (NA = 0.30) objective coupled to a Nikon Eclipse E800 brightfield microscope, equipped with a motorized stage, a 3CCD camera (JVC Ltd., UK) and Surveyor imaging software (Objective Imaging Ltd., UK) (Figure 2Ai). After digitally converting the image to grey‐scale, brightness and contrast were enhanced for optimal representation using Photoshop software (vCS6, Adobe, USA). Using anatomical atlas data [42], the PPN's grey matter‐white matter boundaries were manually traced onto the image file as a separate digital layer using a digital tablet and pen and Photoshop illustration software (Figure 2Aii). This layer was superimposed onto contiguous (second‐in‐series) sections stained with the cholinergic antibody marker that had been scanned similarly to the LFB‐stained ones. The PPN's grey‐white matter boundaries were traced digitally with Photoshop software, platformed on a digital tablet and interfaced with a digital pen (Figure 2B). For the alternative PPN‐delineation method, the distribution of the ChAT‐stained “Ch5” neurons was encircled. To distinguish the “Ch5” (PPN) from the “Ch6” (LDT) cholinergic populations, we followed published anatomical guidelines [4, 46] and consulted the Allen Adult Human Brain reference atlas (atlas.brain-map.org) [47]. Published guidelines state that the LDT lies medial‐posterior to the PPN, is embedded in the pontine central grey region, and lies between the caudal part of the dorsal raphe nucleus and the parabrachial nucleus and ventral to the caudal part of the ventral periaqueductal grey (PAG). Closely packed cells (<75 μm in‐between adjacent cholinergic neurons' somata) were designated as PPNc, while more dispersed cells (cholinergic neuronal somata being situated >75 μm apart) formed the PPNd. Next, the digital template was superimposed onto corresponding ChAT IHC‐stained sections, enabling consistent PPN boundary delineations to facilitate control versus PD cross‐sample comparisons for cholinergic neuronal stereological counts (Figure 2C). Dotted lines delineate WMFT boundaries. Morphological variation in brainstem anatomy, even with age‐matched controls, precluded the use of control templates from being digitally superimposed on PD cases. Hence, for comparisons between controls and PD cases, the PPN was defined using the methodology based on WMFT boundaries only.

### 2.3 Stereological quantification of cholinergic neurons within the PPN

Investigators were blind to the clinical‐ and demographic details of all cases and controls included in this study (e.g., demographic details such as age at death, biological sex and clinical details such as whether the patient phenotype was tremor/gait dominant) during the stereological measurements. For avoiding potential artifacts and errors that may skew results, unbiased design‐based stereological quantification accounts for variation in morphology, orientation and distribution of three‐dimensional (3‐D) study objects (e.g., neuronal somata) observed in a two‐dimensional (2‐D) single‐plane. First, a cell traversing two tissue sections, due to crysectioning of a tissue sample (“block”) producing adjacently lying serial sections, will mistakenly be counted twice if the counter tallies the cells present on each section (Figure 2Ei). Stereology accounts for this issue by utilizing a “dissector,” which compares cell counts from a sampling frame to that from a reference frame, thereby preventing double counting (Figure 2Eii). Second, if cells cluster towards one side of a tissue block, a section taken from either pole might under‐ or overestimate the total number of cells across the sample. The issue can be offset via systematic, evenly spaced sampling across the region of interest (ROI) (Figure 2Eiii). These mitigations rest on stereology's integration of unbiased counting frames and volume probes in its experimental design. Specifically, stereological computation requires the following input measurements: The sampling frame area that is given as a fraction of the total area of interest (AOI), termed the area sampling fraction (asf), and the height of the optical dissector, that is, the distance between the sampling and reference frame, expressed as a fraction of the total section thickness, referred to as the height sampling fraction (hsf). The total estimated cell count per tissue section is then calculated as: *N* = *asf* ^−1^ × *ssf* ^−1^ × *hsf* ^−1^ × ∑*Q*, *Q* being the cell count per counting frame, that is summed across all counting frames per section. When applied to evenly spaced sections across an ROI, for example, every 6th section, as was collected in the present study, to represent a section sampling fraction (ssf) of 1/6, the given formulation allows for generating accurate estimated particle counts representative of the whole AOI.

For stereological quantification of the ChAT‐stained neurons (Figure 2D), a 250 μm × 250 μm counting frame size was used, with the asf that was calculated automatically by Image Pro‐Plus software (Media Cybernetics, Inc., USA), a height sampling fraction (hsf) determined by means of the microscope‐mounted microcator (Germany) and ssf that was set to 1/6, ensuring that every 6th section was stained for the same antigen. Stereological parameters, including section thickness and counting frame dimensions, were selected based on previously published methodologies developed in our laboratory [17, 19]. Although these had originally been applied to rodent PPTg brain tissue sections, we found that they were similarly suitable for interrogating the PPN in human brain tissue, with a coefficient of error (CE) and coefficient of variance (CV) that were calculated for each measurement. In this regard, the overall CE for control subjects was 0.078, the total CV was 0.27, while the overall CE^2^/CV^2^ ratio was 0.08. For the PD cohort, the overall CE was 0.19, the CV was 0.38 and the CE^2^/CV^2^ ratio was 0.26. The Cavalieri principle was used to calculate the volume of the ROI: Areas from all outlined sections were summed and then multiplied by the distance between sections (120 μm) [48].

### 2.4 Three‐dimensional reconstruction model and neuronal counting plots

**FIGURE 3:**
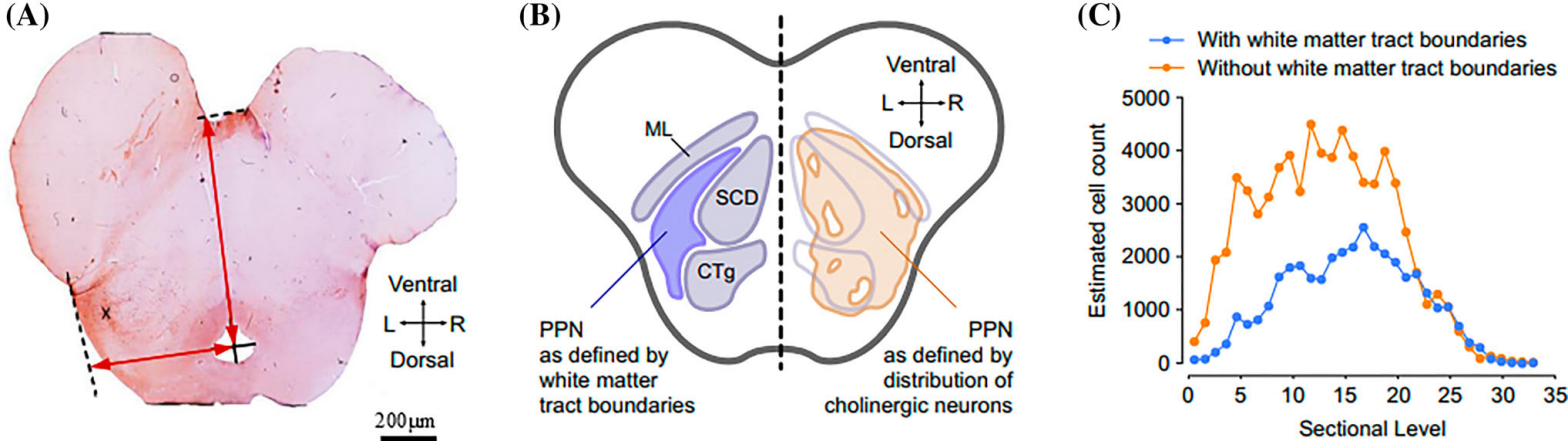
The importance of accurate location of PPN anatomical boundaries to aid sample cross‐comparison studies. (A) A photomicrograph revealing ChAT‐ir neurons within a rostral brainstem tissue section taken in the axial/transverse orientation from a representative neurological‐control patient's post‐mortem brain. For producing a reliable means of localizing the PPN when histological staining is not possible (e.g, for MRI images), the position of the PPN (marked by X), relative to the maximal tegmental dimensions (shown as red axis lines), was determined. The “origin” of these axes was centered on the AqS, a readily identifiable anatomical feature visible on MRI images. The cerebral peduncles were excluded from this measurement due to wide morphological variation, even within neurological‐control brains. The mean M‐L distance from the midpoint of the AqS to the PPN was 66% ± 1.5% of the maximal tegmental width. Similarly, the PPN located at approximately 20% ± 2.6% of the distance between the AqS to the ventral tegmental border. (B) A schematic demonstrating the relationship between the PPN and prominent surrounding white matter tracts, namely the CTg, SCP and ML. Locating the PPN to within the anatomical channel lying between these tracts necessarily lowers the counts of neurons made in this region since many, particularly cholinergic neurons, interdigitate within the traversing tracts. (C) The estimated rostro‐caudal distribution of cholinergic neurons was similar when outlining the PPN according to white matter tract boundaries or the distribution of cholinergic neurons.

Digital templates depicting principal structures in rostral‐caudal sections (120 μm intervals) were taken from representative control and PD cases and aligned using Photoshop software (v.CS6, Adobe, USA). The center of the Aqueduct of Sylvius (AqS), together with the midline of the brainstem, were used as reference points for aligning the images in 3‐D stacks (Figure 3A). The boundary of the PPN was outlined in accordance with previously published guidance [41] as a channel located between the SCP/ML/CTg WMFTs (Figure 3B) and was further recognized as a dense collection of the “Ch5” group of cholinergic neurons [1]. Aligned stacks were imported into Reconstruct software (v.1.1.0.1), using the software's outlining tool to manually trace the PPNc and PPNd structures on each digital image file, each representing a single brain section. Reconstructions were generated using Boissonat surfaces, which were rendered to produce 3‐D models, from which the final volumes of the PPNc and PPNd structures were estimated. Cell count plots were generated in Matlab software (v.R2019, Mathworks, USA).

### 2.5 Data and statistical analyses

All statistical analyses were performed using Prism software (v.8, GraphPad, USA). To statistically compare two group means (e.g., mean cholinergic count in PD versus age‐matched controls), the normality of data distribution per group was assessed using the Shapiro–Wilk test. If one or both groups for paired comparisons failed to pass normality testing, groups were compared using a non‐parametric Mann–Whitney test. Otherwise, where data were Gaussian in distribution, either a two‐tailed or one‐tailed Student's *t* test was performed, depending on the interpretation of the comparison. For all data, differences were considered significant if *p* values were less than 0.05; significance thresholds were as follows: *p* ≤ 0.05*, *p* ≤ 0.01**, *p* ≤ 0.001*** and *p* > 0.05, non‐significant (n/s). Data are expressed as the mean ± standard deviation (SD).

## 3 RESULTS

### 3.1 PPN location can reliably be determined from the position of the inferior colliculus, trochlear nucleus and tegmental dimensions

The PPN's precise neuroanatomical position and boundaries remains debated, with relatively large brain regions set aside to describe the nucleus, the rostro‐caudal dimensions that are typically <5 mm. To delineate the PPN's rostro‐caudal position, we developed a key for predicting the location of the PPN more precisely (Figure 1). The key refers to easily identifiable gross anatomical landmarks, namely the SCP decussation, CTg and the ML, visible under low‐powered (a 5× air‐immersion objective lens) light microscopy applied to unstained histological slides. This reference key was used to interrogate 25 histological sections from the rostral brainstems of control post‐mortem brains and a further 25 slides from post‐mortem PD brains. The samples were taken from tissue that had been sectioned from brainstem‐containing tissue blocks; these glass slide‐mounted tissue sections were available as archived material stored by the Parkinson's UK Brain Bank and had not specifically been sectioned to incorporate the PPN. Thus, the set represented a stochastic process, by randomly containing sections that included the PPN and ones that did not, but in a manner unbeknownst to us. We applied the prediction key (Figure 1) that utilizes information from recognizable gross anatomical features to this set of unstained tissue sections. This resulted in 4 out of 5 accurate identifications of the PPN (80%) in neurological‐control case material; ChAT‐IHC confirmed the presence of the PPN's “Ch5” cholinergic neuronal group. In a single PD case was the PPN's “Ch5” cholinergic neurons were predicted to be visible, based on meeting the anatomical criteria; however, this could not be confirmed via anti‐ChAT IHC staining. Overall, use of the reference key resulted in a 100% sensitivity, a 98% specificity, a positive predictive value of 80% and a negative predictive value of 100%.

As mentioned, the PPN is subdivided into the PPNc and PPNd based on cytoarchitectural and anatomy‐histological distinctions; the PPNc locates within the caudal half of the nucleus and contains mainly cholinergic neurons amassed along the dorsolateral border of the SCP decussation at trochlear nucleus levels that diverge extensively to supply nuclei in the basal ganglia, cerebellum, reticular formation in the lower brainstem and spinal cord [49]. Moreover, the PPNc is a constituent in a feedback loop projecting to the thalamus and serves as a component of the ascending reticular activating system, which enables a state of consciousness. In contrast, the PPNd, located throughout the rostro‐caudal extent of the PPN, mainly contains glutamatergic neurons interspersed with a limited few cholinergic neurons; PPNd glutamatergic projections reach both basal ganglia and spinal cord targets [49, 50, 51].

The different neurochemical profiles and anatomical inputs inherent to the subdivisions indicate functional differences between the PPNc and PPNd, entailing therapeutic targeting consequences. When these subnuclei were discerned based on observed cholinergic neuronal density and non‐transgression of WMFT boundaries, the PPNd contained ChAT‐immunoreactive (ir) neurons that were widely distributed within the surrounding white matter regions (Figure 2C). Moreover, ChAT‐ir neurons were located near the periaqueductal gray region, which more closely associates with the LDT nucleus, another rostral brainstem‐based cholinergic structure. This recurring observation, indicating the arbitrary nature of the PPNd's anatomical definition, along with the PPN‐cholinergic neuronal loss that PD evokes, prompted us to apply anatomical boundaries to identify the PPN for more reliable numerical comparison between control and PD cases. However, the latter method (Figure 2C), by outlining the “Ch5” population's cytochemical boundary, was limited by not being able to include the whole PPN, as much of the PPNd lies within the traversing WMFTs.

For quantifying cholinergic neurons in control subjects, both boundary definitions were used, yielding two values, one representing a more complete value for the entire PPN, the other representing those neurons found in the anatomical channel commonly described as the PPN in atlases. With the aim of identifying the PPN in a clinical setting, for example, when using MRI imaging, a description of the PPN's position in axial sections was sought, but this proved difficult due to variation in brainstem morphology at this level. In particular, the cerebral peduncles showed significant variation in their morphological appearance, even between age‐matched controls. It was therefore impossible to apply an absolute measurement system with consistent results between subjects. To mitigate this, maximal tegmental dimensions (discounting the cerebral peduncles) were evaluated for the PPNc section that contained the most numerous (i.e., the modal value) cholinergic neurons. In each case, the AqS was treated as the point of origin for the axes projecting along the midline and perpendicular to it. PPN measurements (μm) were made from the AqS and expressed as a percentage of the maximal tegmental width (Table 2).

**TABLE 2:** Use of a predictor key for accurate identification of the PPN's position in rostral brainstem tissue sections.

Case identifier	Cerebral hemisphere	Tegmental dimension from the AqS		PPNc's M‐L position (expressed as % of the tegmental M‐L dimension)	PPNc's D‐V position (expressed as % of the tegmental D‐V dimension)
		M‐L (μm)	D‐V (μm)		
PD1	L	8414	12,590	69%	14
	R	8360	12,590	60%	28
PD2	L	12,613	22,534	62%	23
PD3	R	9209	18,129	63%	9
	L	8396	18,129	67%	21
PD4	R	9002	14,761	69%	26
	L	8810	14,761	69%	22
		9258	16,213	66% ± 4%	20% ± 7%

*Note*: The summarized data demonstrates that the PPN's rostro‐caudal position can be accurately predicted as a percentage of the maximum dimensions of the brainstem‐based tegmentum, that is, the PPN typically locates at a 66% traversal from the AqS along the M‐L axis and a 20% traversal from the AqS along the D‐V axis. Combined application of the predictor key and the given % measures renders confidence for predicting the position of the PPN on either MRI‐acquired images or glass‐mounted histopathologic specimens. Data provided in the bottom summary line are expressed as the mean ± SD.

Abbreviations: AqS, Aqueduct of Sylvius; D‐V, dorsoventral; L, left; M‐L, mediolateral; ML, medial lemniscus; PPNc, pedunculopontine nucleus pars compacta; R, right.

During histological processing, formalin diffuses through the tissue, binding to amino groups that precipitate the formation of a network of cross‐linked proteins and nucleic acids, which may cause histological changes, such as cell shrinkage. Few studies have systematically studied the effects of fixation and other histological processes on specimen size; reports of formalin‐induced shrinkage effects in a range of specimens from different organ systems are inconsistent, with some studies reporting sizeable shrinkage [28, 41] while others showed no shrinkage post‐fixation [29, 39]. To mitigate against any such potential effects on our counting paradigms, we expressed the PPN's mediolateral (M‐L) and dorsoventral (D‐V) as a percentage (i.e., a ratio) of the maximal tegmental dimension, our rationale being that processing‐induced tissue shrinkage may affect the fidelity of the absolute measurements taken here, but have only a negligible impact on relative distances, as the effects of shrinkage on the tissue section, if present, can be expected to be relatively uniform across its dimensions. Hence, by expressing the PPN's M‐L position as a proportion of the maximal tegmental width, the metrics given here should be unaffected by any possible tissue shrinkage, as both the numerator and denominator for calculating the percentage are affected by the same shrinkage factor.

In this regard, the mean M‐L distance from the midpoint of the AqS to the PPN was 66% ± 2% of the maximal tegmental width. Similarly, the use of this predictive key placed the PPN at 20% ± 3% of the distance from the AqS to the ventral tegmental border. Thus, the PPN's rostro‐caudal location can be ascertained with a high degree of confidence by using the key given in Figure 1.

### 3.2 PPN‐cholinergic neurons are more abundant than previously reported for neurological‐control brains, with substantial neuronal loss affecting PD brains

We utilized an optimized method to determine cholinergic neuronal numbers within the PPN, using brain tissue sections spanning the full rostro‐caudal extent of the PPN, and by applying two complimentary neuroanatomical approaches for identifying PPN boundaries. Stereological analysis of ChAT‐ir neurons of brain sections containing the PPN, outlined by referencing the “Ch5” cholinergic population [33], taken from 4 neurological‐control cases (thereby sampling seven complete PPNs, irrespective of laterality), revealed a cumulative mean neuronal count of 72,458 ± 5629, that is, ~3.7× of what was previously reported [38]. PD reduced this neuronal population by 48% to 37,383 ± 4565, compared to controls (****p < 0.0001).

However, as stated previously, neuronal quantification depends on the precise boundaries employed in this morphologically heterogenous brain region. Hence, when the traversing WMFTs were employed as PPN boundaries instead, the average unilateral PPN‐cholinergic neuronal count of control brains was 35,842 ± 769. PPN‐cholinergic neuronal quantification using this PPN delineation method resulted in a mean unilateral PPN‐cholinergic cell count in PD brains of 17,936 ± 1905, again resulting in a highly significant statistical difference when compared to counts made in control brains (****p < 0.0001) and correlating with a 50% PD‐induced loss of cholinergic neurons.

**FIGURE 4:**
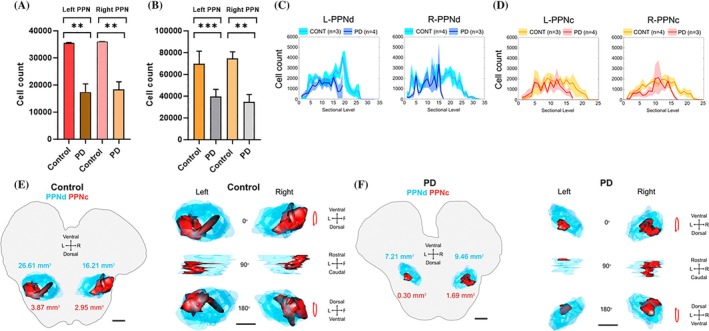
PPN‐cholinergic neurons' bilateral rostro‐caudal distribution with 3‐D PPN reconstruction. (A) PD versus control case comparisons of cholinergic stereological cell counts aided by WMFT boundary delineations of the PPN showed a highly significant loss of cholinergic neurons in PD brains in both left‐ and right‐sided brain hemispheres (~50%, **p < 0.01). (B) Defining the PPN in accordance with its resident “Ch5” cholinergic population also showed PD‐induced depletion equating to ~50%, the loss recorded for the left PPN that was slightly more significant (***p < 0.01) than that for the right‐sided PPN (**p < 0.01). The latter method permitted defining the PPNd separately from the PPNc. In left‐sided PPNs of neurological‐controls, cholinergic neuronal density was exceedingly low at (C) the rostral and (D) caudal poles, while a striking low density was also seen at the PPNd‐PPNc border. In intact PPNs, left–right hemisphere symmetry was conserved. (C, D) Asymmetric neuronal loss was statistically non‐significant; however, a strong trend was observed in some PD cases. Although the rostro‐caudal spatial distribution pattern broadly resembled that of the controls for both hemispheres, the cholinergic neuronal topography was exaggerated in the PD‐affected PPNs. Prominently, in PD specimens, the bimodal peaks that featured for both hemispheres in the intact PPN's rostral half (PPNd) was reduced to a single peak, while the caudal tail of the nucleus was significantly protracted. (E, F) Digital templates of the cholinergic neuronal distribution were lofted to produce a 3‐D representation of the PPN, comprising its PPNc (red) and PPNd (blue) subdivisions. Right‐sided panels provide rotated visualizations of the subnuclei for a representative control and PD case, in either a ventro‐dorsal or rostro‐caudal dimension, as indicated by the information key. (E) A non‐neurogenerative (control) PPN specimen is shown (the left panel is the overview image, the right panel shows detail), showing a complex morphology which highlights the diffuse distribution of its resident cholinergic neurons. Morphologically, the nucleus appears asymmetric across the midline, which may have a bearing on stereotactic target acquisition during PPN‐DBS, although neuronal counts and rostro‐caudal distribution appear relatively symmetric. (F) In this representative PD case, a left‐sided PPN‐cholinergic neuronal loss predominates; however, this was an inconsistent observation across the PD cohort. Right‐sided PPNds showed greater cholinergic neuronal loss in most PD cases, but exceptions were seen as demonstrated in this individual case, where PPNc neuronal loss was especially prominent.

For control cases, irrespective of the nucleus' boundary definition (WMFT‐ or Ch5‐based), the PPN's cholinergic neuronal density appeared to be largely symmetric across the midline (Figure 3C). Defining the PPN's boundary by means of the juxtaposed WMFT‐based PPN delineation method estimated that, on average, left‐sided PPNs contained 35,567 ± 2980, differing only marginally (1.3%) from cholinergic neuronal density of right‐sided PPNs (36,049 ± 1498) (*p* > 0.05, n/s) (Figure 4A). In contrast, when relying on the PPN's boundary definition relating to the distribution of the Ch5 cholinergic neuronal population, left‐sided PPNs contained 69,648 ± 11,715 cholinergic neurons, compared to the right‐sided count of 74,565 ± 6230 (*p* > 0.05, n/s) (Figure 4B). This figure likely represents an upper limit of the true neuronal count and incorporates large numbers of ChAT‐ir cells found interdigitating within nearby traversing WMFTs (Figure 2B,C), whereas it is plausible that a portion of these neurons do not associate functionally with the PPN. Thus, it may be best to consider the PPN's residential cholinergic population count as a range rather than a single value; the importance of representative values highlights the complex architecture of the nucleus with respect to nearby WMFTs, and for quantifying the impact due to PD‐related neurodegeneration.

Cholinergic neuronal hemispheric symmetry appeared to be largely maintained during PD; left‐sided PPNs (*n* = 4) of PD‐affected brains averaged 17,380 ± 3031 (WMFT method) or 39,466 ± 6703 (Ch5 population method) (Figure 4A), showing only marginal cholinergic density differences (5%) compared to the right‐sided PPNs (*n* = 3) (18,354 ± 2824) (WMFT method); 4%; 34,606 ± 7019 (Ch5 population method) (Figure 4B). Paired analysis between right versus left‐sided PPN‐cholinergic neuronal counts derived from PD‐affected brains, compiled using either of the two PPN anatomical delineation methods, revealed no statistically significant difference (*p* > 0.05, n/s).

PD manifests notably asymmetrically, particularly in the earlier disease stages [12]. Here we explored whether the PPN's cellular architecture, especially the density of its remaining cholinergic population, may relate to such asymmetric symptomology. Although our interhemispheric comparison results reveal that PPNs in either cerebral hemisphere are relatively equally affected during PD, and that one cerebral half does not harbor a higher “spare” inherent PPN‐cholinergic population in control brains, possibly relating to patients' hand‐use dominance, the findings require validation using larger left–right hemispherically paired PPN sets per individual PD patient versus control case.

### 3.3 PD‐induced deviations in PPN‐cholinergic rostro‐caudal and hemi‐lateral topographical distribution patterns

The human PPN, oriented along the long axis of the brainstem, consists of two subdivisions, namely the PPNd, comprising the nucleus' rostral half, and the PPNc that lies within the PPN's caudal half. PPN subnuclei, differentially recognized by distinct neuronal distribution patterns, have been described in rats as well as Old and New World monkeys [1, 17, 43, 50, 52]. Here we stereologically profiled human non‐diseased PPN‐cholinergic neurons' rostro‐caudal distribution to comparatively determine how this pattern may be disrupted by PD. Cohort averaged cell counts paired to the rostro‐caudal section‐mounted slide number are shown in Figure 4C,D.

Stereological quantification of IHC‐identified cholinergic neurons revealed a largely symmetric inter‐hemispheric distribution of cholinergic neurons within the PPNd of control cases (left: 43,778 ± 8146, right: 49,226 ± 5552; *p* > 0.5, n/s), with the PPN's rostral domain that returned a mean cholinergic neuronal count across the midline of 46,891 ± 4159. Interhemispheric comparison of cholinergic neurons of the PPNc in control cases also revealed a largely symmetric distribution (left: 25,870 ± 3610, right: 25,339 ± 2497). The mean unilateral (irrespective of laterality) PPNc cholinergic neuronal count was 25,567 ± 1912, similar to previously estimated PPN‐cholinergic neuronal counts (26,443 ± 3289) but which used a profoundly different counting paradigm than the stereological method we employed for the current study [40].

Compared to the PPNc, the PPNd held the bulk of cholinergic neuronal somas (44.9% more; ***p = 0.0004). Regression analysis performed between the sectional area of the PPNc versus PPNd returned an *R* ^2^ value of 0.37 and a *p*‐value of 0.001. This strong correlation trend suggests that the more amorphous PPNd is anatomically (and possibly functionally also) related to the PPNc cluster, while also validating the given boundary definitions. We observed sharp distinguishing borders of cholinergic neuronal densities between the PPNd and PPNc, indicating that the bipartite rodent PPTg model repeats for the human PPN [1, 17]. However, close inspection of these two main peaks revealed small, distinct neuronal clusters potentially linked to functional domains, for example, motor, limbic or associative functions. In this regard, a study attributed function to neuronal density variation in the subthalamic nucleus, postulating that lower neuronal density may represent motor function output [53].

The left‐sided PPNd displayed a bimodal distribution, the initial neuronal density peak being slighter than the second, spanning section (S)20–50 (600 μm) and reaching its apex at S38 (720 μm from rostral‐end; 2158 ± 231 cholinergic neurons). The caudal part of the PPN (S164–278) showed reduced cholinergic neuronal density (25,752 ± 138) compared to the PPNd (S2–158; 43,897 ± 189; 41.3%) (*p* = 0.1834, n/s). The PPNc's general cholinergic neuronal density shape also differed from the bimodal PPNd, instead showing a single central bulk of cells that gradually reduced density towards the caudal boundary.

The rostro‐caudal density distribution of the right‐sided control PPN largely resembled the left‐sided PPN, but with some variation: The bimodal peaks were more well defined in the left hemisphere, with the right‐sided PPN tending towards a more multimodal distribution of such neurons. This applied particularly to the PPNd, with more numerous neuronal density micropeaks and the apex manifesting earlier (S68/1320 μm from rostral‐end), followed by a steeper decline in cholinergic neuronal numbers than seen for the left‐sided PPNd (S110/2160 μm from rostral‐end). The lowest cholinergic neuronal density typically occurred further from the rostral edge (S194/3840 μm) for the right‐sided than for the left‐sided PPN (S158/3120 μm). The right‐sided PPNd (S2–194/3840 μm) was generally more elongated compared to the left‐sided PPNc (S1–27/2120 μm). The right‐sided PPNc was also more well defined with a clearer single central bulk than left‐sided ones, but were of similar lengths (left: S164–278/2280 μm; right: 200–320/2400 μm). For control cases, higher left–right inter‐subject variation was seen for PPNd cholinergic neuronal counts, with less inter‐hemispheric variation noticed in PPNc cholinergic cell counts, the respective CVs being 23.5% and 19.8%.

### 3.4 Rostro‐caudal and hemi‐lateral topographical distribution of cholinergic neurons in non‐PD control PPN specimens compared to PD‐affected PPNs

Severe PPNd‐cholinergic neuronal reduction was seen in PD cases (21,285 ± 3619; 55%; ***p < 0.001), compared to control cases. In PD brains, left PPNd mean cholinergic neuronal counts were estimated at 22,310 ± 5651, compared to right‐sided PPNd estimates of 19,918 ± 5115, representing a 10.7% difference (*p* > 0.05, n/s), to render the disease cohort's left–right PPNd's remaining cholinergic counts slightly asymmetric with a left‐sided bias, opposite to that of control brains that instead revealed an 11% right‐sided PPNd cholinergic density bias. The right‐sided PPNd was worse affected by PD‐induced cholinergic neuronal loss (60%) compared to the left‐sided PPNd (49%), relative to the unihemispheric sides in control cases.

PPNd PPN‐cholinergic neuronal loss was 18% higher than the PD‐induced inter‐hemispheric cell loss that affected the PPNc, the latter which was reduced by 37% to 16,098 ± 1904 compared to control cases (**p < 0.01). The PD‐induced left‐sided PPNc's cholinergic neuronal loss was less pronounced (34%; 17,156 ± 2953 neurons) than right‐sided PPNcs (42%; 14,688 ± 2073), compared to the respective hemispheric halves of control samples. Interhemispherically, PD‐affected PPNcs showed less overall inter‐subject cholinergic neuronal count variation compared to PD‐affected PPNds, the respective CVs being 31% and 45%. Right‐sided PPNcs showed the least cholinergic count variance (CV = 29%), while left‐sided PPNds revealed the most (CV = 51%). In summary, the PPNd was proportionally more affected by cholinergic neuronal reduction due to PD than the PPNc, the right‐sided PPNd's losses that were generally more pronounced. However, considerable case‐by‐case variation was seen as revealed by the calculated CVs. Such inter‐individual variation may relate to both symptom spectrum variation and symptom manifestation laterality. However, a lack of available case notes and a somewhat limited set of available per patient paired left–right hemispheric PPN samples precludes us from confirming this assumption.

Rostro‐caudal topographical assessment of the PD‐affected PPN samples revealed that cholinergic neuronal losses were most pronounced at the polar ends, indicating relative preservation of the central bulk (approximate to the PPNc's location) of both left‐ and right‐sided PPNs. Although a PPNd versus PPNc distinction remained visible as a sharp neuronal reduction between the bimodal neuronal clusters, the pronounced gap in‐between the neuronal bulks seen in the control specimens, were microstructurally altered in PD specimens. A rostro‐caudal section‐by‐section investigation revealed finer detail, hinting at structure–function reorganization of the PPN to possibly preserve cholinergic output during PD‐induced cholinergic atrophy: In left‐hemisphere control samples, the highest neuronal density occurred within the second bimodal peak of the PPNd (~S110/2160 μm from rostral‐end), contrasting with PD specimens' highest neuronal spike, at ~S86 (1680 μm from rostral‐end). The right‐sided PD‐affected PPNs also displayed altered rostral‐to‐caudal topography for this neuronal type; however, neuronal organization appeared more caudally directed, contrasting with the rostral directionality seen for the left‐hemispheric PPN‐cholinergic neurons: Whereas in right‐sided control specimens the sectional domain with the highest neuronal density occurred at S68, this was present at S86 in PD samples. Awareness of such pathology‐induced neurotype‐specific microlesions promises to deliver more optimized neurosurgical targeting of the nucleus for providing effective symptomatic relief to PD patients, by stimulating more neuronally preserved and therefore functionally intact subzones.

### 3.5 Three‐dimensional reconstruction of the PPNc and PPNd subdivisions in control and PD cases

The changes impacting upon the PPN's overall morphology, evident from the stereological evaluation, were also expressed by means of a 3‐D reconstruction of the tissue templates used to generate the cholinergic neuronal count estimates. A single representative case for the control (Figure 4E) and PD group (Figure 4F) was used to generate these models, by connecting contiguous 2‐D tissue templates in an image stack to produce a PPN representation given in 3‐D format. These models visualize the extent of degeneration affecting the PPN of PD patients, while also illustrating how poorly suited data derived from existing human brain atlases are in acquiring the PPN stereotactically, based as they are on control subjects and hence the intact PPN. Volumetric values derived from the stereological analysis, and which reflect in the 3‐D model, revealed that the bilateral PPNs (encompassing both the PPNd and PPNc) of this PD case showed, on average, a 2.5× reduction for the right‐sided PPN, but only a 1.3x reduction for the left hemisphere one, thereby strengthening the case in favor of asymmetric cholinergic neuronal loss during PD, that is slightly skewed (10%) towards a greater right‐sided PPN neuronal loss (and hence nuclear volumetric reduction), as was revealed in our stereological analysis. Unfortunately, lack of clinical detail precludes association of this asymmetry with a precise clinical phenotype.

Three‐dimensional rendering also illustrates interhemispheric and cross‐cohort differences for the PPN's subnuclei which tallied with cohort‐specific cholinergic neuronal changes, but most importantly, serves to illustrate the profound alteration of the PPN's morphology due to PD‐related neuronal loss, thereby greatly distorting the intended nucleus' targeting during DBS.

## 4 DISCUSSION

The current study generated comprehensive stereological data on the number of cholinergic neurons for both the whole PPN and by distinguishing the PPNd from the PPNc subnucleus. Our data further distinguished between advanced PD and control cases, due to evidence that degeneration of PPN‐cholinergic neurons associates with the progressive manifestation of motor and sleep‐related deficits in PD patients [54]. We used two contrasting PPN border definitions as templates for the stereological neuronal counts. PPN neurons reside in continuity with those constituting the reticular formation [55], highlighting the PPN's diffuse nature. Hence, a consistent evaluation of the PPN's inherent cholinergic neuronal population in healthy controls and when affected by PD depended on setting clear nucleus boundary definitions; however, this was challenging owing to a lack of consensus in the published literature as to the PPN's anatomical margins. Previously published neurotype‐specific counts of the PPN utilized the traditional boundary of the PPN based on its surrounding WMFTs [19, 34, 42, 50]. Although several studies reported extensive cholinergic neuronal loss within the PPN in clinical PD (Table S1), none employed a rigorous unbiased stereological method combined with PPN anatomy boundary considerations. Reports using non‐stereological/semi‐stereological counting paradigms cite cholinergic neuronal losses between 36% and 85% [16, 38, 56]. The large range may partly relate to the differential extent of disease, but more likely relates to inconsistent delineation of the PPN's anatomical boundaries and suboptimal sampling strategies for obtaining the neuronal estimates.

The realization that the PPN's anatomical definition is arbitrary and that PD‐induced PPN‐cholinergic neuronal loss renders the PPNc‐PPNd boundary indistinct, prompted us to apply two complimentary anatomical boundary definitions for outlining the nucleus to facilitate more accurate cross‐sample numerical comparisons between control and PD cases. This was done by mapping the PPN via traditional anatomical criteria versus defining the PPN via the “Ch”‐based denomination put forward by Mesulam and colleagues [33]. For neurological‐control brains, the average unilateral PPN‐cholinergic neuronal count was ~72,458 when quantifying this cell group based on the PPN's “Ch5”‐based delineation, compared to ~35,842 when defining the PPN boundaries based on its traversing WMFTs. However, the latter approach is limited as it cannot include the whole PPN since much of the PPNd lies within the traversing WMFTs. Nevertheless, application of either nucleus boundary definition resulted in a closely matched cholinergic neuronal reduction affecting PD‐affected compared to control PPNs, at 48% for the “Ch5”‐based method and 50% for the WMFT method.

Despite intense research efforts to improve description of the PPN's anatomy in both health and when affected by neurodegenerative disease (Table S1), human‐based investigations have largely been superseded by anatomical studies in rodents, for which human correlations remain unevaluated. Specifically, rodent PPN anatomy is hallmarked by the presence of cell clusters or “gradients,” cholinergic neurons being more populous caudally (the PPNc), while GABAergic ones are more numerous rostrally (the PPNd). It has been postulated that such clusters constitute functional specialization within the nucleus [1, 25, 49]. Speculation exists as to whether such gradients repeat itself in human PPNs, how PD may disrupt this topographical organization, and whether these may be specifically targeted via DBS to produce a more refined stimulation outcome. The current report is the first to describe neurotype‐specific rostro‐caudal gradients in post‐mortem PPNs of confirmed PD cases, compared to aged neurological‐controls. In non‐PD PPNs we identified a rostrally biased cholinergic subpopulation that outnumbered PPNc‐based cholinergic neuronal counts by nearly double, a profile that reflected symmetrically across the midline. The rostral‐to‐caudal arranged concentration of cholinergic neurons described here for the human, non‐diseased PPN seems to be evolutionarily conserved, paralleling that described for the pedunculopontine tegmental nucleus (PPTg), the rodent equivalent of the PPN, such studies having speculated that differential functional subspecializations exist between neuronal clusters arranged in the rostral versus caudal half of the nucleus [1, 17]. Further comparative evaluations between the rodent, non‐human primate and human PPN's cellular arrangements using a uniform stereology‐based cell counting paradigm will be insightful for confirming whether differential rostro‐caudal cellular gradient patterns are evolutionarily conserved. In addition, our study, having stratified the data according to cerebral hemispheric half, is a useful template for future work focused on whether the nucleus' cellular distribution pattern is conserved “across the midline” for different species. Future studies should aim to expand the sample set above the size that the current study was able to source for the paired left–right hemisphere PPN subnuclei‐cholinergic neuronal counts, to allow for drawing potentially more meaningful conclusions as to PPNd versus PPNd cholinergic neuronal bilaterality in human and non‐human species.

In PD cases, cholinergic stereological count data plotted along the PPN's rostral‐to‐caudal axis, inspection of the neuronal cluster gradients revealed that in both hemispheres, cholinergic neurons were most compromised at the polar ends of the nucleus, particularly towards the rostral‐end, while the region corresponding to the PPNc was relatively spared. By evaluating the proportional loss attributable to each subnucleus, it is evident that most PD‐induced cholinergic neuronal loss occurs within the PPNd rather than the PPNc, contrasting with stereological studies that utilized toxin‐evoked rodent models of PD [1, 5, 16, 17, 25, 45, 57].

Several studies showed that PPN‐cholinergic neurons are particularly susceptible to PD's disease processes, including a tendency to form intraneuronal α‐synuclein protein aggregates, constituting a neuropathological hallmark of PD [58]. However, debate continues as to the causes, extent, and functional consequences of PPN neuronal loss seen in PD‐affected brains. The PPN lies outside the basal ganglia but has increasingly been associated with basal nuclei functions. Moreover, the PPN has major output projections to the thalamus, the spinal cord and dopaminergic midbrain, specifically to the SNpc, the latter output that is implicated as the most direct modulator of PD‐related symptoms in the context of PPN‐DBS. Studies showed that PPN‐cholinergic neurons can drive action potentials in SNpc dopaminergic neurons, the latter being highly vulnerable to apoptotic events during PD [33, 59]. It remains unconfirmed whether DBS applied to the PPN attains its clinical effects by stimulating its remaining cholinergic neurons [5, 20], or whether PPN‐DBS inference restores excessive GABA‐mediated inhibition of the anterior PPN, which characterizes PD‐affected brains [7]. Our previous work, using a well‐characterized rat model, revealed that a more refined DBS targeting strategy to exclusively modulate PPN‐cholinergic neurons may represent a therapeutic platform to greatly improve axial‐related PD‐like symptoms, possibly avoiding unwanted non‐cholinergic‐associated clinical side effects due to global PPN‐DBS [5, 20]. This effect remains unconfirmed for clinical brains; however, our current results suggest that the efficacy of PPN‐DBS for controlling certain PD symptoms depends critically upon the location of the stimulation implant, which should consider rostro‐caudal cholinergic neuronal clusters and the extent by which such neural distribution patterns are disrupted by PD. This aligns with other human‐based studies that reported varied clinical benefits, depending on whether electrodes were implanted within the caudal [60, 61] or rostral PPN [2, 26, 31, 48].

Our findings further highlight a need to better consider the dimensions of stimulating electrodes for delivering DBS that average >1 mm. In line with others [62], our data support the recommendation of using finer electrodes than currently the case in neurosurgical practice. This will not only limit the potential for damaging the PPN due to mechanical damage and localized heating but also allow for more precise implants for targeting specific neurotype clusters to either dampen/excite their neural outputs in line with a patient's clinical profile and the known neurochemical basis of such symptomology. Electrode technologies that allow for real‐time adjustments during surgery could include electrodes capable of electrical and perhaps chip‐based high‐performance liquid chromatography chemical monitoring. This will permit monitoring chemical processes at the smallest dimensions to allow for biofeedback‐based adjustment of the anatomical position of the electrode and/or DBS stimulation parameters.

Taken together, the current study is the first comprehensive quantification of PPN‐cholinergic neurons in human post‐mortem brains, comparing PD‐affected PPNs to neurological‐control specimens. However, our study has potential limitations which future follow‐up research should consider. Our study was conducted using “gold standard” stereological principles to allow us to accurately estimate PPN‐cholinergic neuronal counts to compare PD‐affected brains to non‐PD control ones. However, our interpretation of the level of left–right hemisphere PPN‐cholinergic neuronal loss due to PD was restricted due to the available sample number and should be repeated using a more expanded sample set to allow for greater confidence in the hemispherically‐paired dataset trends observed here. Moreover, a larger dataset will allow for better reflecting the extent of variability of these counts between the cerebral hemispheres. Although several neurodegenerative conditions present with unilateral symptom onset and development, asymmetric symptom onset by PD patients is especially prominent [3], with their presentation often becoming bilateral as the disease progresses, but with the initially afflicted side that remains worse affected throughout disease duration [12]. An interesting suggestion arising from our preliminary inter‐hemispheric stereology data is whether PD's impact on PPN‐cholinergic neuronal loss laterality may associate with specific hemi‐lateral profiles relating to specific symptoms, for example, increased freezing of gait and/or falls [5, 20, 63, 64]. Future studies should include such correlations using well validated clinical profile data. Such work holds potential translational importance since PD‐induced exacerbation of cholinergic density asymmetry may invalidate coordinates derived from standard stereotactic atlases that are constructed from unilateral specimens and further contribute to the variation described for PPN‐DBS clinical results [2, 15, 18, 22, 23, 31, 44, 48, 65, 66].

The current data holds potentially important ramifications for refining DBS approaches for alleviating PD symptoms. Since most PD cases arise sporadically, manifesting in aged patients (>65 years of age), we compared aged PD to aged neurological‐control cases here. However, PD may occur in younger patients, that is, <65 years of age, especially due to rare familial forms of the disease. The findings reported here may not be translational to such younger PD cases. To establish whether similar cholinergic neuronal profiles apply to non‐aged but PD‐affected PPNs, future work should aim to contrast PPN samples from early‐onset PD cases with non‐aged neurological‐control cases. Findings from such work will enlighten us on the combined impact of normal ageing and PD on the PPN's cholinergic population and determine whether PPN‐targeting DBS in younger PD patients should follow similar targeting guidelines as in aged PD patients.

## AUTHOR CONTRIBUTIONS

Puneet Kumar Sharma, David Trevor Dexter and Ilse Sanet Pienaar coordinated the project and obtained funding. Puneet Kumar Sharma and Ilse Sanet Pienaar performed the experiments and analyzed the data. Steve Gentleman provided specialist neuropathological advice. Puneet Kumar Sharma, David Trevor Dexter and Ilse Sanet Pienaar drafted the manuscript. All authors commented on the manuscript draft and approved the final version.

## CONFLICT OF INTEREST STATEMENT

The authors declare no conflicts of interest.

## Supporting information


**Table S1.** A summary of post‐mortem human studies (arranged chronologically from most recent to earliest) that reported on PPN neuronal quantifications performed on neurological‐control versus PD cases. In some instances, post‐mortem brain material derived from only control cases were analyzed while some investigations had co‐analyzed samples from neurodegenerative diseases other than PD patients. Studies that applied a stereological approach for analyzing neurons within sectioned tissue taken from post‐mortem human PPNs are shaded, while those that utilized a non‐stereological method are unshaded. ACh, acetylcholinesterase; AD, Alzheimer's disease; CGS, central grey substance; CFV, cresyl fast violet; ChAT, choline acetyltransferase; CTg, central tegmental tract; DLB, dementia with Lewy bodies; FoV, fields of view; H&E, hematoxylin and eosin; IC, inferior colliculus; IHC, immunohistochemistry; ir, immunoreactive; LBs, Lewy bodies; LDT, laterodorsal tegmental nucleus; LFB, Luxol fast blue; NADPH, nicotinamide adenine dinucleotide phosphate; NFTs, neurofibrillary tangles; NOS, nitric oxide synthases; PPNc, PPN pars compacta; PPNd, PPN pars dissipata; PSP, progressive supranuclear palsy; SCP, superior cerebellar peduncle; SDAT, senile dementia of Alzheimer's type; SD, standard deviation of the mean; SEM, standard error of the mean; SNpc, substantia nigra pars compacta.

## Data Availability

The authors confirm that the data supporting the findings of this study are available within the article.
