# Supplementary material for: Stereological analysis of cholinergic neurons within bilateral pedunculopontine nuclei in health and when affected by Parkinson's disease
Source: Brain Pathol. 2025 Apr 15;35(5):e70011. doi: 10.1111/bpa.70011 (PMC12352924; doi:10.1111/bpa.70011)
Supplement: Supplementary file 1 — Table S1. A summary of post‐mortem human studies (arranged chronologically from most recent to earliest) that reported on PPN neuronal quantifications performed on neurological‐control versus PD cases. In some instances, post‐mortem brain material derived from only control cases were analyzed while some investigations had co‐analyzed samples from neurodegenerative diseases other than PD patients. Studies that applied a stereological approach for analyzing neurons within sectioned tissue taken from post‐mortem human PPNs are shaded, while those that utilized a non‐stereological method are unshaded. ACh, acetylcholinesterase; AD, Alzheimer's disease; CGS, central grey substance; CFV, cresyl fast violet; ChAT, choline acetyltransferase; CTg, central tegmental tract; DLB, dementia with Lewy bodies; FoV, fields of view; H&E, hematoxylin and eosin; IC, inferior colliculus; IHC, immunohistochemistry; ir, immunoreactive; LBs, Lewy bodies; LDT, laterodorsal tegmental nucleus; LFB, Luxol fast blue; NADPH, nicotinamide adenine dinucleotide phosphate; NFTs, neurofibrillary tangles; NOS, nitric oxide synthases; PPNc, PPN pars compacta; PPNd, PPN pars dissipata; PSP, progressive supranuclear palsy; SCP, superior cerebellar peduncle; SDAT, senile dementia of Alzheimer's type; SD, standard deviation of the mean; SEM, standard error of the mean; SNpc, substantia nigra pars compacta. [file BPA-35-e70011-s001.docx]

**Supplementary Table 1. A summary of post-mortem human studies (a****rranged chronologically from most recent to earliest) that reported on PPN neuronal quantifications performed on neurological control versus PD cases.** In some instances, post-mortem brain material derived from only control cases were analysed whilst some investigations had co-analysed samples from neurodegenerative diseases other than PD patients. Studies that applied a stereological approach for analysing neurons within sectioned tissue taken from post-mortem human PPNs are shaded, whilst those that utilised a non-stereological method are unshaded.

| **Reference** | **Number of cases** | | **Quantification method** | | **Principal findings** |  |
| --- | --- | --- | --- | --- | --- | --- |
| [42] | - Control (*n*=9) - PD (*n*=8) | **Stereological**   - Left hemisphere only - Tissue sectioned at 20µm thickness - PPN-containing sections identified with combined Loyez-CFV IHC applied to the most caudal and rostral aspects of the serial sections - Cholinergic, GABAergic and glycinergic neurons counted using neurotype specific fluorescent IHC - Each neuronal subtype was counted in four PPN sections, spaced three serial sections apart and covering an area of 1.949 mm^2^ - Images captured with a 40x/0.75 objective lens, stitched together with AxioVision software (v. 4.6, Carl Zeiss, Germany) - Counts performed with the cell counter function in ImageJ software (v.1.4, NIH, USA)   Counts are given as number of neurons/mm^2^ ± SD | | - Losses of PPN GABAergic (18%), glycinergic (13%) and cholinergic (50% neurons in PD cases vs controls | | |
| [21] | - Control (*n*=9) - PD (*n*=9) - DLB (*n*=9) | **Stereological**   - Right hemisphere only - Tissue sectioned at 20µm thickness - Tissue blocks collected at the level of the IC and pons - Every 10^th^ section was Thionin-stained to delineate the PPNc borders - ChAT IHC identified cholinergic neurons - Neurons were counted via the optical fractionator method followed by 3-D reconstruction - Neuronal density estimates given as number of cells/mm^3^ | | - PPNc cholinergic cell density was significantly lower in PD vs DLB patients (-39%) and vs controls (-41%) | | |
| [26] | - Controls (*n=8*) - PD (*n=12*) | **Stereological**   - Unspecified hemispheric sides - Section thickness was not provided, only described as five “regularly spaced” sections - PPN boundaries not discussed, hence the area (or volume) of counting region is unclear | | - PD patients stratified into ‘faller’ and ‘non-faller’ categories - Faller PD cases: 609 ± 61 vs 934 ± 63 neurons in non-faller PD cases (35% reduction); and 1,058 ± 52 neurons in controls (42% and 12% reduction, respectively) | | |
| [49] | - Control (*n*=9) - PD (*n*=11) | **Stereological**   - Left hemisphere only - Tissue sectioned at 11µm thickness - No borders specifically mentioned, but counts made within the PPNc only - Every 40^th^ section was LFB stained; every 41^st^ section was stained for ChAT - Only cells >20µm counted - Stereological counts made using the Abercrombie formula [1], rather than a dissector | | - Mean loss of cholinergic cells in PD vs controls was 36% - 23% loss of non-cholinergic neurons in PD - Greatest reduction was seen in caudal half of the PPNc - Somal shrinkage of cholinergic (~26%) and non-cholinergic neurons (~14%) was seen in PD | | |
| [30] | - Control (*n*=6) | **Non-stereological**   - Unspecified hemispheric sides - Tissue sectioned at 50µm thickness - Counts made every 10^th^/20^th^ section’ (0.5/1mm between sections) using a computer reconstruction of the mapped section - Quantification utilised an arbitrary correction factor for double counting - ChAT-ir neurons counted | | - Combined PPN and LDT counts: 19,400 ± 655 - Cholinergic neuron distribution: 30% PPNc; 57% PPNd and 13% LDT - PPNc cholinergic neurons: 58%; remainder (42%) neurons were non-cholinergic | |  |
| [15] | - Controls (*n=5*) - PD (*n=6*) | **Non-stereological**   - Unspecified hemispheric sides - Tissue sectioned at 50µm thickness, cut coronally with consecutive sections retained - Precise boundaries for the PPTg not given - Stained for SP-ir; counting was stratified as ‘large’ or ‘small’ neurons based on soma measurements being > or <20µm | | - Total SP-ir cells in controls (54465 ± 4493) vs PD (31065 ± 5291): A 43% loss - Large SP-ir cells in controls (36935 ± 3994) vs PD (17038 ± 4710): A 54% loss - Small SP-ir cells in controls (17530 ± 1132) vs PD (13208 ± 1460): A 25% loss | |  |
| [20] | - Controls (*n=4*) - PD (*n=4*) | **Non-stereological**   - Unspecified hemispheric sides - Tissue sectioned at 50µm thickness; 15 sections per series (spanning 750µm) - Originated from a coronally dissected block; PPN boundaries not described - SP-stained cells counted | | - Control PPN SP+ve Count (1,043 ± 235; count ± SEM) vs PD (450 ± 121): A 57% loss | |  |
| [71] | - Control (*n*=8) - PD (*n*=4) - Mixed PD/AD (*n*=4) | **Non-stereological**   - Unspecified hemispheric sides - Tissue sectioned at 12µm thickness - Every 20^th^ section stained with CFV - A single section from three levels along the rostro-caudal axis was counted - Only PPNc neurons counted; boundaries set by SCP and CTg and by CFV staining to identify neuropil - Cells >20 µm counted | | - 46% neuronal loss in PD vs controls - Significant negative correlation between PPNc neuronal count and SNpc neuronal loss - Cell counts varied considerably between PD patients | |  |
| [24] | - Control (*n*=15) - PD (*n*=14) - PSP (*n*=2) - AD (*n*=5) - SDAT (*n*=17) | **Non-stereological**   - Unspecified hemispheric sides - Tissue sectioned at 5-7µm thickness - Samples were from two PPN levels - Only evaluated the PPNc, but delineating borders not described - Subtype non-specific neurons counted in CFV-H&E-stained sections - Cells counted if >20µm and containing prominent nucleoli and abundant Nissl substance - Neuronal counts made under ×400 magnification using an ocular µm - LBs and NFTs counted in the same FoV | | - 66-85% neuronal (neurotype non-specific) loss in PPNs of PD cases - 53% PPNc neuronal loss in PD vs controls - LBs detected in 6-39% PD-affected PPNc neurons; NFTs seen in 0-5% of remaining neurons | |  |
| [22] | - Control (*n*=4) - PD (*n*=6) - PSP (*n*=3) | **Non-stereological**   - One (unspecified) hemispheric side analysed; bilateral analysis made for a single case - Tissue sectioned at 40µm thickness - Variable inter-section quantification intervals: 720µm (8 brains); 200 and 520µm, alternating (3 brains); and 1440µm (2 brains) - ACh and NADPH (co-localised with neuronal NOS) IHC - NADP-ir cell counts made in a reference square within the PPNd and PPNc, excluding the CGS - NADPH cell counts expressed as percentage of control; ACh activity determined by radioenzymatic assay | | - Authors were unable to validate PPN NADP-ir neurons as cholinergic - 57% loss of presumed cholinergic neurons in PD vs controls - Cross-sectional width of the PPNc was reduced by 38% in PD vs controls - PD-affected brains varied i.t.o. PPN neuronal loss: Four cases were moderate-severe whilst two cases revealed marginal neuronal loss | |  |

Abbreviations used: *ACh* Acetylcholinesterase*, AD* Alzheimer’s disease, *CGS* central grey substance, *CFV* Cresyl Fast Violet, *ChAT* Choline acetyltransferase, *CTg* Central tegmental tract, *DLB* Dementia with Lewy Bodies, *FoV* Fields of View, *H&E* Haematoxylin & eosin, *IC* Inferior colliculus, *IHC* Immunohistochemistry, *ir* Immunoreactive, *LBs* Lewy bodies, *LDT* Laterodorsal tegmental nucleus, *LFB* Luxol fast blue, *NADPH* Nicotinamide adenine dinucleotide phosphate, *NFTs* Neurofibrillary Tangles, *NOS* Nitric oxide synthases, *PPNc* PPN pars compacta*, PPNd* PPN pars dissipata, *PSP* Progressive supranuclear palsy, *SCP* superior cerebellar peduncle, *SDAT* Senile Dementia of Alzheimer’s Type, *SD* Standard deviation of the mean, *SEM* Standard error of the mean, *SNpc* Substantia Nigra pars compacta.
